# ALCOHOL USE SCREENING AND INTEGRATED BEHAVIORAL HEALTH IN GERIATRIC PRIMARY CARE IN THE DEEP SOUTH

**DOI:** 10.1093/geroni/igz038.3394

**Published:** 2019-11-08

**Authors:** Bailey Lanai, Deanna Dragan, Rebecca S Allen, Anne Halli-Tierney, Dana Carroll, Amy Albright

**Affiliations:** 1 The University of Alabama, Tuscaloosa, Alabama, United States; 2 The University of Alabama College of Community Health Sciences, Tuscaloosa, Alabama, United States

## Abstract

This longitudinal behavioral health surveillance and integrated care project aims to assess physical and mental health and substance use in a geriatric primary care setting. Approximately 230 patients (mean age = 76; 74% female; 16% African American) attending an interdisciplinary geriatrics clinic in Alabama have taken part in baseline behavioral health screenings since 2014. Behavioral health measures include cognitive status, self-reported mood, subjective and objective health literacy, and alcohol use. All measures are administered by clinical psychology graduate students. Patients had an average of 5.83 medical diagnoses. Only 26.2% of patients had scores indicating cognitive functioning within normal limits; 32.6% had scores indicative of mild neurocognitive disorder, and 41.2% had scores indicative of dementia. Over 80% of patients had adequate self-reported health literacy; however, measurements of objective health literacy indicated a significant number of individuals have difficulty following medical directions independently. Over 30% of patients reported clinically significant levels of depression or anxiety, and 16.5% of patients reported at least one indicator of hazardous alcohol use. Specifically, 50.7% of patients consume alcohol on at least a yearly basis with 38.2% endorsing at least one problematic drinking behavior and 11.6% scoring in the clinically significant range for alcohol misuse. Moreover, 22.7% report use of opioid pain medication. The results of this study demonstrate that routine hazardous alcohol use screening as one component of integrated behavioral health care within geriatric primary care increases detection of hazardous alcohol use among older adults.

